# Editorial Comment: A systematic review of best practices for the perioperative management of abdominal sacrocolpopexy

**DOI:** 10.1590/S1677-5538.IBJU.2021.06.05

**Published:** 2021-10-01

**Authors:** Cássio L. Z. Riccetto

**Affiliations:** 1 Universidade Estadual de Campinas Faculdade de Ciências Médicas Divisão de Urologia Feminina CampinasSP Brasil Divisão de Urologia Feminina, Faculdade de Ciências Médicas da Universidade Estadual de Campinas – UNICAMP, Campinas, SP, Brasil.

Amy Nemirovsky 1, Amber S Herbert 1, Emily F Gorman 2, Rena D Malik 1

Neurourol Urodyn. 2020 Jun;39(5):1264-1275.

DOI: 10.1002/nau.24411 | ACCESS: 10.1002/nau.24411

## COMMENT

The authors aimed to systematically assess the literature according to the PRISMA statement to determine whether there is sufficient evidence to create best practice guidelines for abdominal sacrocolpopexy (ASC). At the end of the selection 35 studies were considered eligible for qualitative analysis. The authors highlighted that laparoscopic and robotic ASC equivalently determined a shorter postoperative hospital stay and that the use of epidural analgesia decreased the level of postoperative pain in laparoscopic ASC. They also pointed out that the early removal of the urethral catheter was significantly associated with higher rates of urinary retention and urinary tract infection. In addition, studies that investigated preoperative intestinal preparation and use of pre-anesthetic medications did not show any significant benefit. The use of combined antibiotic therapy was not superior to the use of a single isolated agent. Finally, a multicenter double-blind study compared liberal versus restrictive recommendations regarding physical activities in the postoperative period of minimally invasive ASC and found no differences in satisfaction and anatomical results after 3 months.

ASC has taken a leading role in the to the treatment of pelvic organ prolapses involving the vaginal apex. However, differently from techniques performed vaginally, laparoscopic and robotic approaches impose greater operative risks (1, 2) in patients that usually display important comorbidities. Such increased risk justifies the effort towards development of perioperative guidelines, which can be very useful in increasing the safety of procedures. Similarly, the mission of Enhanced Recovery After Surgery (ERAS) Society (3) is to develop perioperative care and to improve recovery through research, education, audit and implementation of evidence-based practice.
